# Environmental Factors Influencing the Dynamics and Evolution of COVID-19: A Systematic Review on the Study of Short-Term Ozone Exposure

**DOI:** 10.3390/healthcare11192670

**Published:** 2023-10-01

**Authors:** Irina-Maria Popescu, Luminita Mirela Baditoiu, Sandhya Rani Reddy, Akhila Nalla, Emilian Damian Popovici, Madalin-Marius Margan, Mariana Anghel, Sorina Maria Denisa Laitin, Ana-Olivia Toma, Alexandra Herlo, Roxana Manuela Fericean, Nina Baghina, Andrei Anghel

**Affiliations:** 1 Department of Infectious Diseases, Discipline of Epidemiology, “Victor Babes” University of Medicine and Pharmacy, Eftimie Murgu Square 2, 300041 Timisoara, Romania; irina.stefan@umft.ro (I.-M.P.); baditoiu.luminita@umft.ro (L.M.B.); epidemio@umft.ro (E.D.P.); anghel.mariana@umft.ro (M.A.); laitin.sorina@umft.ro (S.M.D.L.); 2 Doctoral School, “Victor Babes” University of Medicine and Pharmacy, Eftimie Murgu Square 2, 300041 Timisoara, Romania; manuelafericean@umft.ro; 3 Department of General Medicine, Prathima Institute of Medical Sciences, Nagunur 505417, Telangana, India; reddysandhya094@gmail.com; 4 Department of General Medicine, MNR Medical College, Sangareddy 502294, Telangana, India; akhila.nalla@gmail.com; 5 Department of Functional Sciences, Discipline of Public Health, “Victor Babes” University of Medicine and Pharmacy, Eftimie Murgu Square 2, 300041 Timisoara, Romania; margan.madalin@umft.ro; 6 Department of Dermatology, “Victor Babes” University of Medicine and Pharmacy, Eftimie Murgu Square 2, 300041 Timisoara, Romania; 7 Department of Infectious Diseases, Discipline of Infectious Diseases, “Victor Babes” University of Medicine and Pharmacy, Eftimie Murgu Square 2, 300041 Timisoara, Romania; alexandra.mocanu@umft.ro; 8 National Meteorological Administration of Romania, Soseaua Bucuresti-Ploiesti 97, 013686 Bucuresti, Romania; ninabaghina@yahoo.com; 9 Department of Biochemistry and Pharmacology, “Victor Babes” University of Medicine and Pharmacy, Eftimie Murgu Square 2, 300041 Timisoara, Romania; biochim@umft.ro

**Keywords:** COVID-19, SARS-CoV-2, environmental health, ozone

## Abstract

The potential influence of environmental factors, particularly air pollutants such as ozone (O_3_), on the dynamics and progression of COVID-19 remains a significant concern. This study aimed to systematically review and analyze the current body of literature to assess the impact of short-term ozone exposure on COVID-19 transmission dynamics and disease evolution. A rigorous systematic review was conducted in March 2023, covering studies from January 2020 to January 2023 found in PubMed, Web of Science, and Scopus. We followed the PRISMA guidelines and PROSPERO criteria, focusing exclusively on the effects of short-term ozone exposure on COVID-19. The literature search was restricted to English-language journal articles, with the inclusion and exclusion criteria strictly adhered to. Out of 4674 identified studies, 18 fulfilled the inclusion criteria, conducted across eight countries. The findings showed a varied association between short-term ozone exposure and COVID-19 incidence, severity, and mortality. Some studies reported a higher association between ozone exposure and incidence in institutional settings (OR: 1.06, 95% CI: 1.00–1.13) compared to the general population (OR: 1.00, 95% CI: 0.98–1.03). The present research identified a positive association between ozone exposure and both total and active COVID-19 cases as well as related deaths (coefficient for cases: 0.214; for recoveries: 0.216; for active cases: 0.467; for deaths: 0.215). Other studies also found positive associations between ozone levels and COVID-19 cases and deaths, while fewer reports identified a negative association between ozone exposure and COVID-19 incidence (coefficient: −0.187) and mortality (coefficient: −0.215). Conversely, some studies found no significant association between ozone exposure and COVID-19, suggesting a complex and potentially region-specific relationship. The relationship between short-term ozone exposure and COVID-19 dynamics is complex and multifaceted, indicating both positive and negative associations. These variations are possibly due to demographic and regional factors. Further research is necessary to bridge current knowledge gaps, especially considering the potential influence of short-term O_3_ exposure on COVID-19 outcomes and the broader implications on public health policy and preventive strategies during pandemics.

## 1. Introduction

Since the emergence of SARS-CoV-2 and its associated disease, COVID-19, in late 2019, the global scientific community worked tirelessly to understand its transmission dynamics, pathogenesis, and various environmental factors that influence its spread [1,2,3]. As a pandemic that brought the world to a standstill, COVID-19 has highlighted the crucial role of environmental health in disease progression by exacerbating or ameliorating the impacts of the virus on susceptible populations [4,5]. Initially, it was hypothesized that increased levels of pollutants such as fine particulate matter (PM_2.5_), nitrogen dioxide (NO_2_), and ozone (O_3_) could heighten susceptibility to respiratory infections, including COVID-19, and exacerbate disease outcomes [6]. These pollutants are known to cause inflammation and damage to the respiratory tract, potentially amplifying vulnerability to the virus [7]. However, other hypotheses present a more nuanced view, suggesting that elevated environmental O_3_ might play a protective role in reducing respiratory infections and mortality. This dual perspective on the role of O_3_ warrants a detailed exploration, which is what this review seeks to accomplish.

An important environmental factor that has received significant attention in the context of respiratory diseases is ambient air pollution, specifically ozone [8]. Ozone is a potent oxidant, and elevated concentrations in the troposphere have been associated with various health problems, including acute and chronic respiratory diseases [9]. Epidemiological studies have demonstrated a significant association between O_3_ exposure and increased morbidity and mortality, particularly in patients with pre-existing respiratory and cardiovascular diseases [10].

Given the respiratory nature of COVID-19, it is plausible to assume a potential link between ozone exposure and the severity or progression of this viral disease [11]. Environmental ozone could affect host resistance, thus impacting susceptibility to SARS-CoV-2 infection and the subsequent course of the disease [12]. Moreover, the relationship between O_3_ and COVID-19 may be influenced by a variety of factors such as climatic conditions, population density, social behaviors, health infrastructure, and an individual’s health status [13]. Understanding the complex interplay between these factors and COVID-19 infection rates is essential to develop comprehensive strategies for disease prevention and control.

Existing literature presents inconsistent findings concerning the relationship between environmental pollution and COVID-19 outcomes [14,15]. Some studies suggest a positive correlation between ozone exposure and other air pollutants and COVID-19 case rates, whereas others have reported a negative correlation or no correlation [16,17]. The inconsistencies among these studies might be attributed to differences in methodology, the pollutants studied, demographic factors, local weather and environmental conditions, public health measures, population behavior, and the stage of the pandemic in which the studies were conducted [18,19,20,21]. Thus, these inconsistencies underline the importance of a systematic review to collate and critically evaluate existing evidence on this topic.

This study’s hypothesis is that short-term ozone exposure influences the dynamics and evolution of COVID-19. The potential role of ambient O_3_ levels in influencing the susceptibility and outcomes of viral respiratory infections is gradually coming to the forefront. It is assumed that elevated concentrations of ozone in the atmosphere could enhance the vulnerability to SARS-CoV-2 infection, possibly by compromising the respiratory immune response, thus exacerbating the clinical outcomes of infected individuals. Conversely, environments characterized by lower O_3_ concentrations may potentially mitigate the severity and transmission dynamics of the disease, possibly through lessened oxidative stress and irritation in the respiratory tract. However, the exact mechanisms remain the subject of ongoing research. In this systematic review, we aim to explore the existing scientific literature to provide a more comprehensive understanding of these potential relationships and their implications for public health strategies.

Therefore, the current study aims to systematically review and analyze existing literature concerning the effects of short-term ozone exposure, typically characterized as exposure periods ranging from a few hours to several days, on COVID-19 transmission dynamics and evolution. Additionally, it aims to identify gaps in the current knowledge and provide recommendations for future research directions. Such a comprehensive review is necessary to improve our understanding of COVID-19 in the context of air pollution, with potential implications for public health strategies, policy-making, and preventive measures during current or future pandemics.

## 2. Materials and Methods

### 2.1. Protocol and Registration

This systematic review was conducted in March 2023 through an extensive search of three electronic databases: PubMed, Web of Science, and Scopus. We included literature published from January 2020, the inception of COVID-19, until January 2023. The search strategy used medical subject headings (MeSH) keywords such as “COVID-19”, “SARS-CoV-2”, “Ozone”, “Air Pollution”, “Environmental Factors”, “Disease Dynamics”, “Epidemiology”, “Respiratory Disease”, “Disease Evolution”, and “O3 Exposure”. The strategy included the following string: “COVID-19” and “Ozone” OR “Air Pollution” OR “Environmental Factors” OR “Disease Dynamics” OR “Epidemiology” OR “Respiratory Disease” OR “Disease Evolution” OR “O3 Exposure”.

This review strictly adhered to the Preferred Reporting Items for Systematic Reviews and Meta-Analyses (PRISMA) guidelines [22] and the International Prospective Register of Systematic Reviews (PROSPERO) criteria [23]. We employed a structured and systematic search strategy to identify relevant scientific papers investigating the impact of short-term ozone exposure on the dynamics and evolution of COVID-19. This systematic review was registered on the Open Science Framework (OSF) platform [24].

The central research question aimed to determine the effects of short-term ozone exposure on the dynamics and evolution of COVID-19. As part of our exploration, we sought to answer several sub-questions, including the association between short-term ozone exposure and COVID-19 susceptibility, disease severity, and mortality, and the potential for environmental O_3_ levels to modulate the course of the disease and its outcomes.

### 2.2. Eligibility Criteria

The literature search was limited to English-language journal articles. The selection process started with the removal of duplicate entries, followed by a comprehensive evaluation of each abstract performed by two independent researchers to assess their relevance to the research questions. The bibliographies of full-text publications were examined for additional potentially relevant studies; a practice known as cross-referencing. Afterward, a meticulous review of the full text was carried out for the remaining articles to ensure that they met the inclusion criteria.

The inclusion criteria for this systematic review were: (1) studies addressing the impact of short-term ozone exposure on the dynamics and evolution of COVID-19; (2) clinical outcome measures including, but not limited to, COVID-19 infection rates, severity of symptoms, mortality rates, and disease progression; and (3) detailed description of the methods used to measure and classify short-term ozone exposure. Conversely, the exclusion criteria were: (1) studies not addressing the effects of short-term ozone exposure on COVID-19 dynamics and evolution; (2) studies lacking relevant data on clinical outcomes; (3) articles in which the methods for measuring short-term ozone exposure were not explicitly described; and (4) in vitro studies, case reports, proceedings, reviews, commentaries, and letters to the editor.

### 2.3. Data Collection Process

The initial search yielded a total of 4674 studies, from which duplicates were identified and removed. After excluding irrelevant papers based on their abstracts, two authors meticulously examined the remaining full-text articles for relevance. A third author performed a triple check to ensure thoroughness and accuracy. Ultimately, 18 articles were deemed eligible for inclusion in the systematic review.

We used the Quality Assessment Tool for Observational Cohort and Cross-Sectional Studies to evaluate the included articles. Each question within the tool received a score of 1 for “Yes” responses and 0 for “No” and “Other” responses. This scoring system was used to determine the final quality score for each study. Studies with scores from 0 to 4 were labeled as poor quality, those scoring between 5 and 9 were labeled as fair quality, and those with a score of 10 or above were deemed high quality. To minimize bias and enhance reliability, two researchers independently assessed the quality of the selected articles.

### 2.4. Risk of Bias

**Figure 1:**
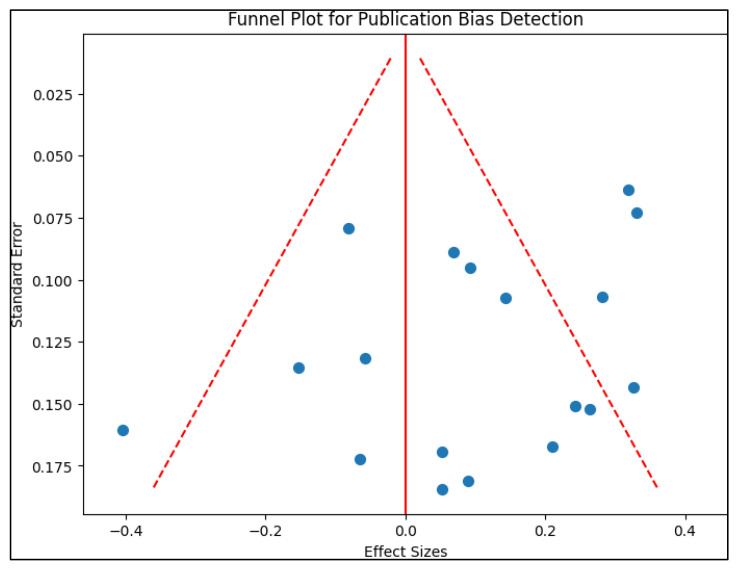
Funnel plot for publication bias.

Publication bias was assessed by creating a funnel plot (Figure 1), where the standard error of the log odds ratio was plotted against its corresponding log odds ratio. The symmetry of the plot was visually examined and further assessed using Egger’s regression test, with a *p*-value < 0.05 indicating significant publication bias. A sensitivity analysis was also performed by removing one study at a time and recalculating the pooled odds ratios. This process aimed to evaluate the robustness of the results and examine the impact of individual studies on the overall effect size.

## 3. Results

### 3.1. Study Characteristics

**Figure 2:**
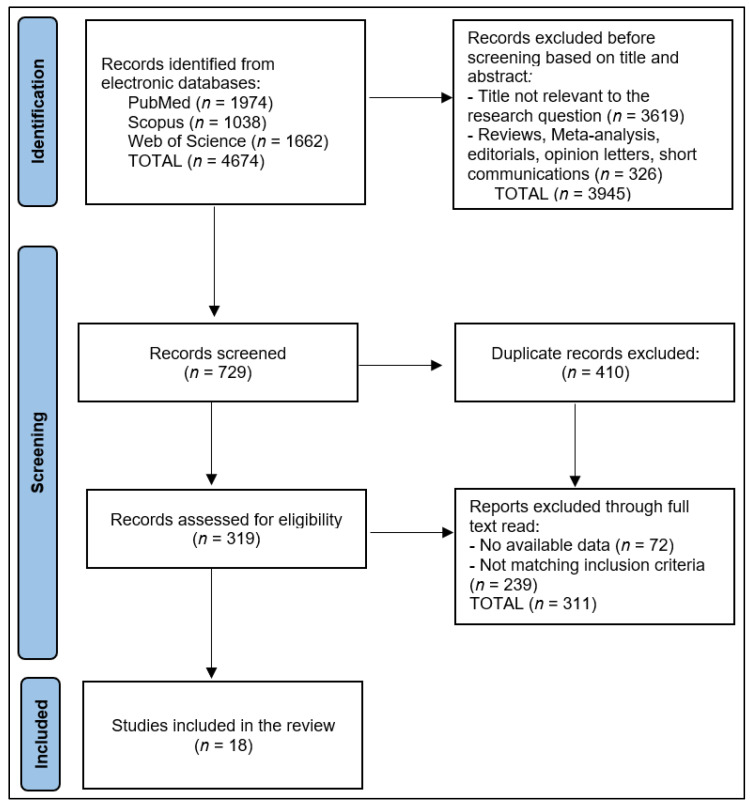
PRISMA flow diagram.

This systematic review included 18 studies [25,26,27,28,29,30,31,32,33,34,35,36,37,38,39,40,41,42] that investigated the influence of environmental factors, specifically short-term ozone exposure, on the dynamics and evolution of COVID-19, as presented in Figure 2. These studies were conducted across eight countries—Canada, Germany, Italy, Mexico, Spain, the UK, the USA, and Turkey—indicating a widespread international concern and interest in this line of research. All the studies were conducted within the context of the COVID-19 pandemic, emphasizing the importance and urgency of understanding the relationship between ozone exposure and the progression of the virus.

The countries involved in the research span different continents, demonstrating a global response towards understanding the influences of the environment on COVID-19. In Europe, the studies were conducted in Germany [26,27], Italy [28,29,30], Spain [32], the UK [33], Poland [42], and Turkey [41]. In North America, the studies were conducted in Canada [25], Mexico [31], and across several states in the USA [34,35,36,37,38,39,40]. This broad geographical spread illustrates the universal relevance of this research area.

Regarding the study designs, the majority of the studies employed a retrospective observational design [26,27,28,29,31,33,35,36,41,42], four studies employed a time series analysis [25,30,32,34,38], one study used a longitudinal analysis [39], and one study used a case-crossover analysis [40]. This diversity of study designs provided a comprehensive exploration of the research question.

An evaluation of study quality revealed that the studies were predominantly characterized as ‘Good’ [26,28,29,32,33,34,37,38,39], with a few studies rated as ‘Excellent’ [25,27,36,40,41] and ‘Fair’ [30,31,42]. The excellent-quality studies were led by researchers like To et al. [25] and Kim et al. [40], displaying robust design and rigorous statistical analysis. The good-quality studies provided consistent and reliable findings, while the ‘Fair’ studies, despite some limitations, contributed to the overall understanding of the research question. This distribution of ‘Excellent’, ‘Good’, and ‘Fair’ studies ensures a comprehensive and varied investigation into the impact of short-term ozone exposure on COVID-19 dynamics (Table 1).

### 3.2. Study Design and Outcomes

This systematic review also assessed the study designs and outcomes, as shown in Table 2. In all 18 studies, the common outcome was the daily incidence of COVID-19 cases, providing a consistent measure to assess the influence of ozone exposure and other pollutants. Several studies further expanded their scope to include outcomes such as daily deaths [27,30,31,33,34,37,41], total cases [26], total deaths [26], daily prevalence rates [28], and rates of emergency admission [32]. These additional outcomes provided a broader picture of the impacts of environmental factors on COVID-19.

In addition to ozone, many studies examined the impacts of other pollutants such as particulate matter (PM_2.5_ and PM_10_) [26,28,29,31,32,33,34,36,41], nitrogen dioxide (NO_2_) [26,28,30,31,32,41], carbon monoxide (CO) [28,33,34], sulfur dioxide (SO_2_) [28,29,31,41], and ammonia (NH_3_) [28]. These additional pollutants allowed for a comprehensive evaluation of the potential synergistic effects between ozone and other common air pollutants.

Various statistical models were used across the studies, highlighting the diverse and robust approaches applied in the analysis of the data. These ranged from generalized linear models using restricted maximum likelihood [25,32], Spearman correlation [26,33,34,41,42], instrumental variable for air pollution using region-specific daily variation in wind direction [27], Pearson correlation [28,30], univariable mixed model with a logarithm transformation [29], Poisson regression analysis [33,34], difference in differences with fixed effects [35], generalized additive models (GAMs) and machine learning ensemble-based dynamic emission models (EDEMs) [36], negative binomial regression models and hurdle regression [37], spatio–temporal multivariate time series models [38], linear regression modeling with covariates [39], and case-crossover analysis [40].

### 3.3. Study Results

As described in Table 3, To et al. [25] suggested that while ozone had a limited association with the incidence of COVID-19 in the general population, it was more significantly related to the incidence in institutional settings such as long-term care homes and hospitals. Conversely, Bilal et al. [26] identified a positive association between ozone exposure and both total and active COVID-19 cases as well as related deaths.

Isphording et al. [27] found no statistically significant correlation between acute ozone exposure and COVID-19 cases or deaths, but they reported a positive effect of PM10 on cases and deaths in individuals over 60 years old. Dragone et al. [28] found no significant correlation between ozone and COVID-19 based on a spatial analysis, but they reported strong correlations with other pollutants like PM_2.5_, PM_10_, NH_3_, and CO. In contrast, Stufano et al. [29] reported no evident relationship between ozone and COVID-19 cases, noting inconsistencies depending on the specific lag period considered.

Zoran et al. [30] found a positive and statistically significant association between ozone and total COVID-19 cases, incidence, and total deaths. Similarly, Kutralam-Muniasamy et al. [31] found a positive association between ozone and COVID-19 cases and deaths. However, Linares et al. [32] and Meo et al. [33,34] demonstrated more nuanced results, suggesting regional variations in the impact of ozone and noting demographic factors that might influence the severity of COVID-19 cases.

**Figure 3:**
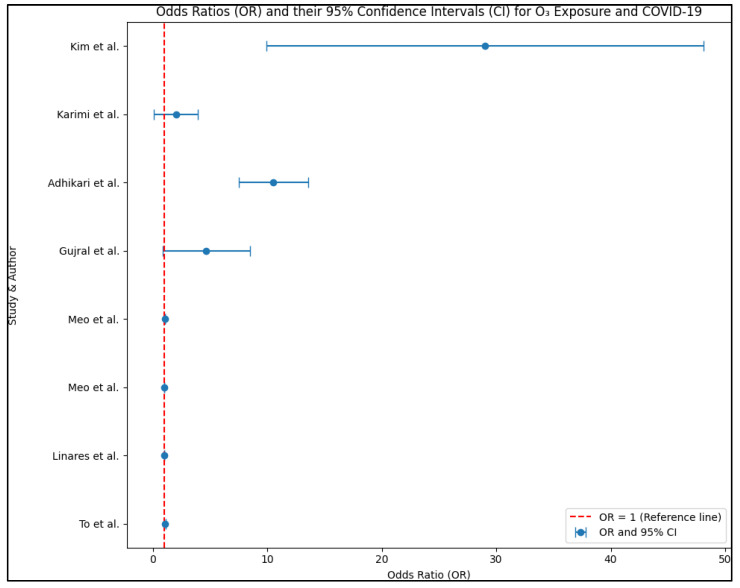
O_3_ relationship with COVID-19 across the analyzed studies [25,32,33,34,36,37,39,40].

In studies by Persico et al. [35], Gujral et al. [36], and Adhikari et al. [37], positive associations between ozone levels and both cases and deaths from COVID-19 were found. In contrast, Rui et al. [38] reported that the total atmospheric ozone density was negatively associated with the incidence of cases, and Karimi et al. [39] found a decrease in COVID-19-related deaths when the average ozone concentration was increased, as presented in Figure 3.

**Figure 4:**
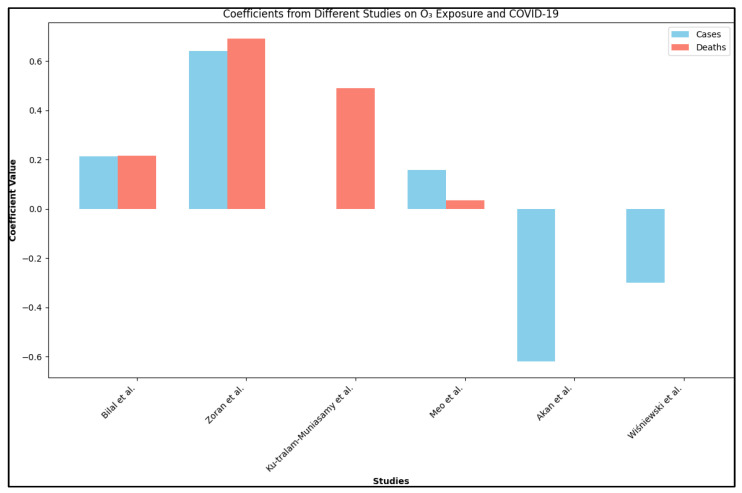
Coefficients of O_3_ exposure and COVID-19 [26,30,31,35,41,42].

Furthermore, Kim et al. [40] highlighted a demographic dimension to these effects, noting variations based on race/ethnicity and comorbid conditions. Lastly, Akan et al. [41] and Wiśniewski et al. [42] reported a decrease in the number of reported cases with increased O_3_ concentrations, as seen in Figure 4. Therefore, the relationship between short-term ozone exposure and COVID-19 dynamics is complex, demonstrating both positive and negative associations with the incidence and severity of the disease. These variations are likely due to demographic and regional factors.

## 4. Discussion

### 4.1. Literature Findings

The impact of environmental factors on the dynamics and evolution of COVID-19, particularly the role of ozone exposure, has been extensively studied, yet the findings remain inconclusive. A systematic review of the current research presents a nuanced picture, emphasizing the complexity of the interaction between ozone levels and COVID-19 transmission, hospitalization, and mortality rates.

The body of literature reviewed here reveals a complex and multifaceted relationship between O_3_ levels and COVID-19 incidence and outcomes, with studies reporting a range of findings—from positive associations to negligible or even negative correlations. It is essential to delve deeper into this complexity to potentially unravel the underlying mechanisms that govern these observed relationships. The positive association between O_3_ levels and COVID-19 cases or deaths, as reported in studies by To et al. [25], Bilal et al. [26], Meo et al. [33,34], and Kim et al. [40], might hint at an underlying biological mechanism wherein elevated O_3_ levels could affect respiratory health or immune responses adversely, thus making individuals more susceptible to the virus. This theory might align with known detrimental health effects of ozone exposure, particularly in the respiratory system.

However, this narrative is contrasted by other studies, such as those by Isphording et al. [27], Dragone et al. [28], and Stufano et al. [29], which suggest an inconsistent or negligible association between O_3_ levels and COVID-19 outcomes, indicating that other confounding factors might be at play. It might also be worth exploring if different methodologies or regional variations could be contributing to these diverse findings. Interestingly, the studies by Akan et al. [41] and Wiśniewski et al. [42] propose a potential protective role of higher O_3_ concentrations, which seems counterintuitive, but opens avenues for exploring novel perspectives or mechanisms in O_3_–COVID-19 dynamics. Given these conflicting narratives, it appears that a one-size-fits-all conclusion regarding the role of O_3_ in the spread and severity of COVID-19 may not be feasible at this stage.

Compared to other pollutants, the role of ozone in influencing COVID-19 outcomes appears less certain. While some studies showed that PM_2.5_, PM_10_, and NO_2_ were significantly associated with COVID-19 cases and deaths [26,28,31,32,35,37,39], the findings on ozone were mixed. Controversially, other authors found that increased environmental O_3_ would reduce the infectivity and even mortality of COVID-19 [41,42]. A possible reason for these conflicting results might be the variation in methodological approaches, geographic locations, and population characteristics across different studies.

Several reasons might explain these inconsistent findings across studies. These could include differences in study design, study settings (geographical and seasonal variability), population characteristics, variations in measuring and categorizing air pollution exposure, as well as controlling for confounding factors such as weather conditions, population density, mobility, and healthcare accessibility, among others. Moreover, the biological mechanisms linking ozone exposure and COVID-19 outcomes are yet to be clearly elucidated. Ozone is a powerful oxidant known for its deleterious health effects, including pulmonary inflammation and impaired immune function, which might render individuals more susceptible to respiratory viruses such as SARS-CoV-2. However, more research is needed to establish this relationship conclusively.

In many regions, elevated levels of ozone are primarily a consequence of vehicular emissions. These heightened ozone levels have been historically associated with an increase in respiratory ailments including, but not limited to, asthma. This backdrop makes the potential inverse correlation between high surface ozone levels and COVID-19 fatalities particularly intriguing, hinting at a possible protective effect that warrants further investigation. It is important to note that ozone is not just a component of the atmosphere; it also finds applications in municipal and healthcare settings owing to its potent sanitizing properties. In municipal contexts, it serves as a critical agent in water purification systems, helping in maintaining hygiene and preventing the spread of water-borne diseases. In healthcare environments, ozone is used as a sterilizing agent, aiding in maintaining sterile conditions and potentially reducing the risk of healthcare-associated infections.

Furthermore, recent laboratory experiments have hinted at another promising facet of ozone: its potential capability to neutralize the SARS-CoV-2 virus [43]. This finding could pave the way for innovative strategies in managing the spread of COVID-19, possibly including the development of ozone-based sanitization protocols to reduce viral transmission in community and healthcare settings. Given the varied roles of ozone, both as a potential risk factor in respiratory health and a tool in sanitization practices, a comprehensive understanding of its multifaceted interaction with COVID-19 dynamics becomes crucial. Future research should aim to dissect these complex relationships further, exploring both the risks and benefits associated with ozone exposure in the context of the COVID-19 pandemic.

Different literature sources indicated an inverse crude correlation between the average ozone concentration in large Chinese cities between January and March 2020 and confirmed cases of COVID-19 [44]. Furthermore, a global study reported a negative link between COVID-19 transmission rates and ozone levels, but the exposure was long-term compared to the short-term exposure evaluated in the current systematic review [45].

Other research has uncovered a noteworthy association between short-term exposure to traffic-related air pollution (TRAP), specifically PM_2.5_, NO_2_, and CO, and extended recovery periods in COVID-19 patients [46]. This aligns with prior studies linking COVID-19 incidence and severity to these air pollutants [47]. For instance, exposure to PM_2.5_ in the recent past appeared to heighten the risk of delayed recovery, potentially due to the particles’ propensity to enter the body through the respiratory tract and accumulate over time. This theory was supported by an earlier Dutch study, which identified PM_2.5_ as a risk factor for COVID-19 development [48].

An interesting theory proposed by some researchers is that particulate matter might also facilitate the transmission of the virus [49]. Notably, there were traces of the COVID-19 virus found in certain types of environmental matter. Traffic and fuel emissions, known producers of NO_2_, could have chronic impacts on human cardiovascular and respiratory systems, and one study found a significant correlation between NO_2_ and COVID-19 health and mortality indicators.

In addition to ozone, other studies observed a positive association between COVID-19 incidence and NO_2_ levels, with an increase in the latter correlating with a nearly 7% rise in daily diagnosed cases [26]. However, the regional distribution of COVID-19 coincided substantially with areas of high pollutant concentrations. Despite this, some studies showed no correlation between NO_2_ and an increase in COVID-19 cases, pointing to the need for further investigation into the relationship between TRAP and COVID-19 risk [50].

### 4.2. Study Limitations

The studies reviewed here have limitations which could influence their results. Many of these studies are observational and cross-sectional, meaning that they can only suggest an association rather than prove a cause-and-effect relationship. Also, measurements of pollutants are usually made in outdoor air and may not accurately reflect personal exposure, as individuals spend a large proportion of their time indoors.

The present systematic review had several limitations that must be acknowledged. Primarily, the diversity of the variables considered in the different studies incorporated into this review could have introduced some heterogeneity into the findings. For instance, while all the studies assessed the impact of short-term ozone exposure on COVID-19, many also included other pollutants like particulate matter, nitrogen dioxide, carbon monoxide, sulfur dioxide, and ammonia, which could potentially have interacted with ozone in ways not accounted for in our analysis, making it challenging to isolate the direct effects of ozone exposure. Moreover, the studies included in this review used diverse study designs and statistical models, which further contributes to the heterogeneity of the results.

Our review did not deeply explore the nuanced interplay between comorbidities or risk factors and O_3_ exposure. Understanding these interactions could have offered additional insights into the varying impacts of O_3_ exposure on different population subsets. While our systematic review intended to provide a broader picture of O_3_’s impact on COVID-19, the absence of this detailed analysis is a limitation. Another notable limitation was the absence of a meta-analysis, which might have given a more quantitative and conclusive insight into the relationship between O_3_ exposure and COVID-19 dynamics. Instead, our approach was primarily qualitative, aggregating and synthesizing results from different studies. This approach, while comprehensive, might not provide the same depth of analysis that a meta-analysis would offer.

Lastly, the studies compiled in our review covered diverse regions, methodologies, and additional pollutants alongside O_3_. This diversity might have introduced heterogeneity, making it challenging to delineate the specific and direct effects of short-term O_3_ exposure on COVID-19. The concurrent evaluation of other pollutants like particulate matter, nitrogen dioxide, carbon monoxide, sulfur dioxide, and ammonia in some studies might influence the outcomes, as their interaction with ozone was not fully addressed in our synthesis.

## 5. Conclusions

In conclusion, there is a growing body of evidence suggesting a possible link between short-term environmental pollutants and COVID-19 outcomes. While there is some evidence suggesting that short-term high O_3_ levels might be associated with increased rates of COVID-19 cases, hospitalizations, or deaths, the specific role of ozone remains uncertain, as other studies have described contradictory findings. Future research should compare short-term and long-term exposure to ozone and focus on conducting well-designed longitudinal studies to establish causality. It is also recommended to further explore the biological mechanisms underlying the potential effects of ozone on COVID-19. Given the pressing nature of the COVID-19 pandemic and widespread exposure to environmental pollutants, understanding these interactions may have significant implications for public health interventions and policy making.

## Figures and Tables

**Table 1:** Study characteristics.

Study and Author	Country	Study Design	Study Quality
1 [25] To et al.	Canada	Time series analysis	Excellent
2 [26] Bilal et al.	Germany	Retrospective observational	Good
3 [27] Isphording et al.	Germany	Retrospective observational	Excellent
4 [28] Dragone et al.	Italy	Retrospective observational	Good
5 [29] Stufano et al.	Italy	Retrospective observational	Good
6 [30] Zoran et al.	Italy	Time series analysis	Fair
7 [31] Kutralam-Muniasamy et al.	Mexico	Retrospective observational	Fair
8 [32] Linares et al.	Spain	Time series analysis	Good
9 [33] Meo et al.	UK	Retrospective observational	Good
10 [34] Meo et al.	USA	Time series analysis	Good
11 [35] Persico et al.	USA	Retrospective observational	Good
12 [36] Gujral et al.	USA	Retrospective observational	Excellent
13 [37] Adhikari et al.	USA	Time series analysis	Good
14 [38] Rui et al.	USA	Time series analysis	Good
15 [39] Karimi et al.	USA	Longitudinal analysis	Good
16 [40] Kim et al.	USA	Case-crossover analysis	Excellent
17 [41] Akan et al.	Turkey	Retrospective observational	Excellent
18 [42] Wiśniewski et al.	Poland	Retrospective observational	Fair

**Table 2:** Study design and outcomes.

Study and Author	Other Pollutants	Study Outcome	Statistical Model
1 [25] To et al.	NR	Daily incidence of cases; reproductive number	Generalized linear models using restricted maximum likelihood.
2 [26] Bilal et al.	$PM_{2.5}$; $PM_{10}$; $NO_{2}$	Total cases; total deaths; prevalence of cases; number of recovered patients	Spearman correlation; wavelet transform coherence approach.
3 [27] Isphording et al.	$PM_{10}$	Daily incidence of cases; daily deaths	Instrumental variable for air pollution using region-specific daily variation in wind direction.
4 [28] Dragone et al.	$PM_{2.5}$; $PM_{10}$; NO; $NO_{2}$; CO; $SO_{2}$; $NH_{3}$	Daily incidence of cases; daily prevalence rate; growth factor	Pearson correlation; time series analysis for each province separately
5 [29] Stufano et al.	$PM_{2.5}$; $PM_{10}$, $SO_{2}$; $NO_{2}$	Daily incidence of cases	Univariable mixed model with a logarithm transformation.
6 [30] Zoran et al.	$NO_{2}$	Daily incidence of cases; total number of cases; daily deaths	Pearson correlation
7 [31] Kutralam-Muniasamy et al.	$PM_{2.5}$; $PM_{10}$; $SO_{2}$; $NO_{2}$; CO	Daily incidence of cases; daily deaths	Correlation analysis (not specified)
8 [32] Linares et al.	$PM_{10}$; $NO_{2}$; Saharan dust	Daily incidence of cases; rate of emergency admission	Generalized linear models with Poisson link.
9 [33] Meo et al.	$PM_{2.5}$; CO	Daily incidence of cases; daily deaths	Spearman correlation; Poisson regression analysis.
10 [34] Meo et al.	$PM_{2.5}$; CO	Daily incidence of cases; daily deaths	Spearman correlation; Poisson Regression Analysis; Binary Logistic Regression.
11 [35] Persico et al.	$PM_{2.5}$	Daily and weekly incidence of cases; daily deaths	Difference in differences with fixed effects.
12 [36] Gujral et al.	$PM_{2.5}$; $PM_{10}$	Daily incidence	Generalized additive models and machine learning ensemble-based dynamic emission model.
13 [37] Adhikari et al.	$PM_{2.5}$	Daily incidence of cases; daily deaths	Negative binomial regression model and hurdle regression.
14 [38] Rui et al.	NR	Daily incidence of cases	Spatio–temporal multivariate time series models
15 [39] Karimi et al.	$PM_{2.5}$	Daily deaths	linear regression modeling with covariates
16 [40] Kim et al.	$PM_{2.5}$	Daily deaths	Case-crossover analysis
17 [41] Akan et al.	$PM_{10}$; $NO_{2};\;PM_{2.5};\;SO_{2}$	Daily incidence of cases; daily deaths	Spearman correlation
18 [42] Wiśniewski et al.	NR	Daily incidence of cases	Spearman correlation

NR—not reported; PM—particulate matter; CO—carbon monoxide; NO_2_—nitric oxide; SO_2_—sulfur dioxide.

**Table 3:** Study results.

Study and Author	Results	Interpretation
1 [25] To et al.	O_3_ risk for hospitalized patients (OR: 1.06 *, 95% CI: 1.00–1.13) O_3_ risk for the general population (OR: 1.00, 95% CI: 0.98–1.03)	Ozone is not significantly associated with incidence nor reproductive number, but it is positively associated with incidence in institutional settings like long-term care homes, hospitals, and jails. A one-unit increase in average weekly ozone is close to being significant for institutional outbreaks but not for the general population.
2 [26] Bilal et al.	O_3_ coefficient for cases: 0.214 * O_3_ coefficient for recoveries: 0.216 * O_3_ coefficient for active cases: 0.467 * O_3_ coefficient for deaths: 0.215 *	PM_10_ and O_3_ are positively associated with total and active cases. The results for PM_2.5_, NO_2_, and cases are mixed depending on whether the outcome is based on active or total cases. O_3_ and NO_2_ are significantly positively associated with COVID-19 deaths. PM_2.5_ is negatively associated with deaths. There is no significant association between PM_10_ and deaths.
3 [27] Isphording et al.	O_3_—no significance PM_10_ 1 μg/m^3^ increase: RR = 1.00042 *	There are significant positive effects of acute exposure to PM_10_ on COVID-19 cases for all individuals and for deaths in those over 60 years old. Similar results were observed for ozone, but the effects were quantitatively non-significant. Among male patients aged 60–79 years, a one μg/m^3^ increase in PM_10_ two to four days after the onset of illness is associated with 0.042 additional deaths per 100,000 individuals. A one-SD increase in air pollution corresponds to an approximately 24 percent of a standard deviation increase in the fatality rate within this demographic.
4 [28] Dragone et al.	PM_10_ > 50 μg/m^3^ PM_2.5_ > 50 μg/m^3^ 75%< RH < 85% 4 °C < AT < 8 °C −0.5 < NAA < 0.5	Based on a spatial analysis, the results indicate that PM_2.5_, PM_10_, NH_3_, and CO are strongly correlated with COVID-19. On the other hand, NO and NO_2_ show weak correlations, while O_3_ and SO_2_ show almost no correlation. However, it is important to note that none of these results reached statistical significance based on the z score values presented in the table.
5 [29] Stufano et al.	NR	In general, there is no evident relationship observed between pollutants and COVID-19 cases. The relationship between the two variables is inconsistent, with both positive and negative associations observed depending on the specific lag period considered.
6 [30] Zoran et al.	O_3_ coefficient for cases: 0.640 * O_3_ coefficient for deaths: 0.690 *	NO_2_ is negatively and statistically significantly associated with total cases, incidence, and total deaths. On the other hand, O_3_ is positively and statistically significantly associated with total cases, incidence, and total deaths.
7 [31] Kutralam-Muniasamy et al.	PM_10_ coefficient for deaths: −0.380 * CO coefficient for deaths: 0.860 * O_3_ coefficient for deaths: 0.490 *	PM_2.5_, NO_2_, and SO_2_ did not exhibit significant associations with cases or deaths. However, PM_10_ displayed a negative association with both cases and deaths. On the other hand, CO and O_3_ showed positive associations with cases and deaths. These findings suggest that higher levels of CO and O_3_ were linked to increased cases and deaths related to COVID-19. The associations observed for PM_10_, CO, and O_3_ were statistically significant.
8 [32] Linares et al.	O_3_ incidence risk (RR: 1.007 *, 95% CI: 1.004–1.009)	In all eight regions analyzed, NO_2_ showed a positive association with COVID-19 cases in terms of incidence rates. Additionally, in six out of the eight regions, NO_2_ displayed a positive association with hospitalizations. Similarly, PM_10_ exhibited a positive association with cases in six regions and hospitalizations in three regions. Furthermore, O_3_ demonstrated a positive association with cases in four regions and hospitalizations in three regions. These findings indicate that air pollutants, especially NO_2_, are closely linked to both the incidence and severity of COVID-19.
9 [33] Meo et al.	O_3_ incidence risk (RR: 1.008 *) O_3_ death risk (RR: 1.044 *)	A 1 μm increase in PM_2.5_ was found to be significantly associated with a 1.1% increase in cases and a 2.3% increase in deaths. Similarly, a 1-unit increase in the CO level is significantly associated with a 21.3% increase in cases and a 21.8% increase in deaths. Furthermore, a 1-unit rise in O_3_ is significantly associated with a 0.8% increase in cases and a 4.4% increase in deaths.
10 [34] Meo et al.	O_3_ incidence risk (RR: 1.025 *) O_3_ coefficient for cases: 0.158 * O_3_ coefficient for deaths: 0.034	The analysis revealed positive associations between PM_2.5_ and CO with both COVID-19 cases and deaths. Additionally, O_3_ was found to have a positive association with cases, but the association with deaths was not statistically significant. Moreover, the results of a Poisson regression indicated that a 1 μm increase in PM_2.5_ resulting from wildfires led to a 0.4% increase in the number of deaths.
11 [35] Persico et al.	NR	Both PM_2.5_ and O_3_ show positive associations with both cases of and deaths due to COVID-19. Specifically, an 11.8 percent increase in PM_2.5_, corresponding to an increase of 0.778 mg/m^3^, is associated with a 53 percent increase in cases. Similarly, a 5 percent increase in ozone is associated with a 10 percent increase in deaths due to COVID-19. These findings highlight the potential impact of air pollution, particularly PM_2.5_ and O_3_, on the incidence and severity of COVID-19 cases.
12 [36] Gujral et al.	O_3_ incidence risk (OR: 4.66, 95% CI: 0.85–8.47)	An increase of one unit in PM_2.5_, PM_10_, and O_3_ is correlated with a decrease of 4.51%, a decrease of 1.62%, and an increase of 4.66% in daily COVID-19 cases, respectively. These findings indicate that higher levels of PM_2.5_ are associated with a decrease in COVID-19 cases, while higher levels of O_3_ are linked to an increase in cases. The effects of PM_10_ on cases is relatively smaller, with a slight decrease observed.
13 [37] Adhikari et al.	O_3_ incidence risk (OR: 10.51 *, 95% CI: 7.47–13.63)	A one-unit increase in the moving average of PM_2.5_ was associated with a 33.11% decrease in daily COVID-19 incidence. On the other hand, a one-unit increase in the moving average of ozone was associated with a 10.51% increase in incidence. Regarding COVID-19 deaths, there was no significant association found with either PM_2.5_ or ozone (O_3_) based on the analysis.
14 [38] Rui et al.	NR	The density of total atmospheric ozone is negatively associated with the incidence of cases.
15 [39] Karimi et al.	O_3_ death risk (OR: 2.0, 95% CI: 0.10–3.60)	The analysis did not find a significant association between PM_2.5_ and COVID-19-related deaths. However, a one ppb increase in the average ozone concentration was associated with a 2.0% decrease in COVID-19-related deaths.
16 [40] Kim et al.	O_3_ death risk (OR: 29.0 *, 95% CI: 9.9–51.5)	A high percentage of the population (98.9%) had ozone (O_3_) levels below the maximum 8 h national ambient air quality standard (NAAQS) of 35.7 μg/m^3^ or 70 parts per billion. An IQR increase in 3-day O_3_ exposure (8.2 μg/m^3^) was associated with a 29.0% increase in the risk of COVID-19 mortality. The associations varied depending on demographics, race/ethnicity, and comorbid conditions, indicating potential modifiers of the observed associations.
17 [41] Akan et al.	O_3_ coefficient for cases: −0.620 *	As the concentration of O_3_ increases, there tends to be a decrease in the number of reported cases of the particular condition under study.
18 [42] Wiśniewski et al.	O_3_ coefficient for cases: −0.299 *	As the concentration of O_3_ increases, there tends to be a decrease in the number of reported cases of the particular condition under study.

*—statistically significant; OR—odds ratio; CI—confidence interval; SD—standard deviation.

## Data Availability

Not applicable.
